# Supplementary material for: Nuclear S-nitrosylation impacts tissue regeneration in zebrafish
Source: Nat Commun. 2021 Nov 1;12:6282. doi: 10.1038/s41467-021-26621-0 (PMC8560954; doi:10.1038/s41467-021-26621-0)
Supplement: Supplementary file 1 — Supplementary Information [file 41467_2021_26621_MOESM1_ESM.pdf]

Supplementary Material for

Nuclear S-nitrosylation impacts tissue regeneration in zebrafish

Gianfranco Matrone, Sung Yun Jung, Jong Min Choi, Antrix Jain, Hon-Chiu Eastwood Leung, Kimal Rajapakshe, Cristian Coarfa, Julie Rodor, Martin A Denvir, Andrew H Baker and John P Cooke

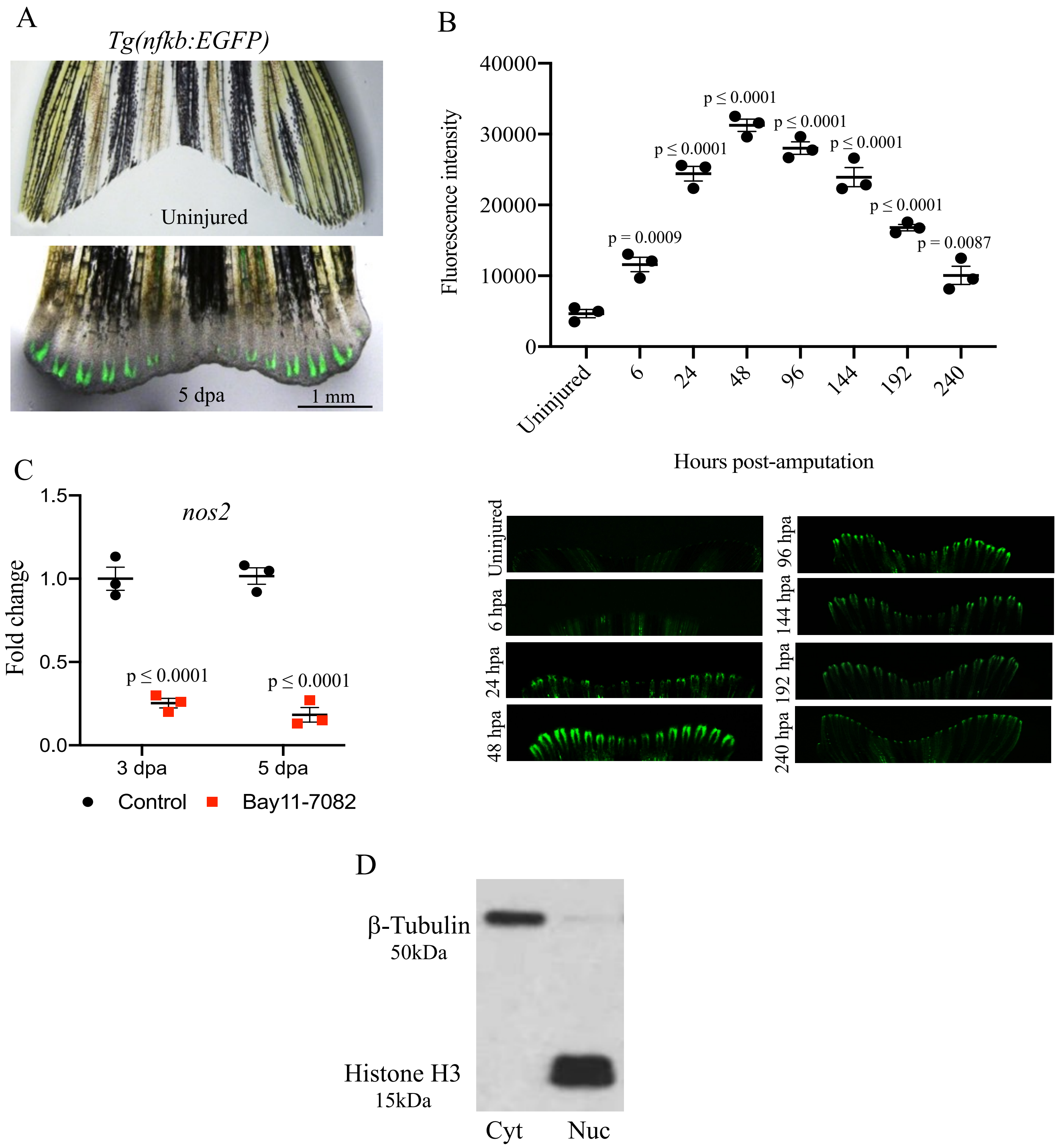

Figure S1 – Activation of the innate immune system following tailfin injury. A. Images (fluorescence and brightfield merged) of the *Tg(nfkb:EGFP)*^{nc1} zebrafish tailfins showing the increase of Nf-kb activity (green fluorescence) following tailfin injury, here shown at 2 days post-amputation (dpa), compared to the uninjured. **B.** Dot plot graph showing the quantification of the fluorescent GFP signal at different time-points after injury. Fluorescent images at the respective time-points are shown in the lower panels. One-way ANOVA test followed by Bonferroni's multiple comparisons test, p values vs uninjured. **C.** Real-time PCR for *nos2* in zebrafish tailfin at 3 and 5 dpa following treatment with the Nf-kb inhibitor Bay11 30 mM and control vehicle. Two-way ANOVA test followed by Bonferroni's multiple comparisons test, p values vs control. **D.** Western blotting showing the effective fractionation of cytoplasmic and nuclear proteins from zebrafish tailfin and blotted with antibodies for β-tubulin and Histone H3, respectively cytoplasmic and nuclear markers. N=3 biological replicates. Data are presented as mean values +/- SEM.

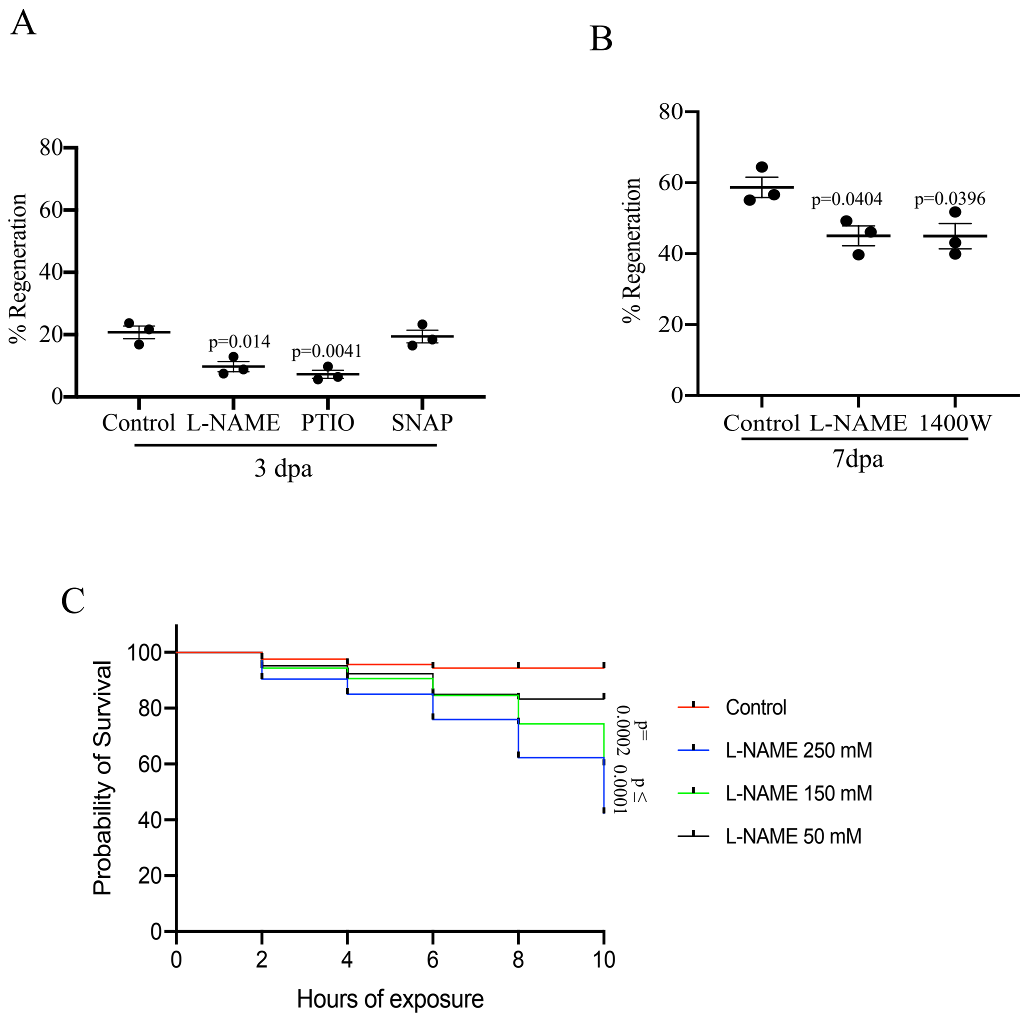

Figure S2– Effects of total Nos and Nos2 inhibition on tailfin regeneration. A. Dot plot showing changes in tailfin regeneration rate at 3 dpa following treatments with L-NAME 50 mM, 2-Phenyl-4,4,5,5-tetramethyl imidazoline-1-oxyl 3-oxide (PTIO) 10 mM, S-Nitroso-N-acetyl-DL-penicillamine (SNAP) 30 mM, compared to control (PBS). **B.** Treatment with 1400W (specific Nos2 inhibitor) 50 mM reduced tailfin regeneration similarly to L-Name (general Nos inhibitor) 50mM, showing that Nos2 is the main Nos isoenzyme involved in the regenerative process. **C.** Kaplan-Meyer survival curve following treatment of adult zebrafish with different doses of L-NAME 50 mM. Log-rank test and Gehan-Breslow-Wilcoxon test were used for statistical analysis of Kaplan-Meyer curve. One-way ANOVA followed by Bonferroni's multiple comparisons test was used for **B** and **C**. p values vs control. N=3 biological replicates. Data are presented as mean values +/- SEM.

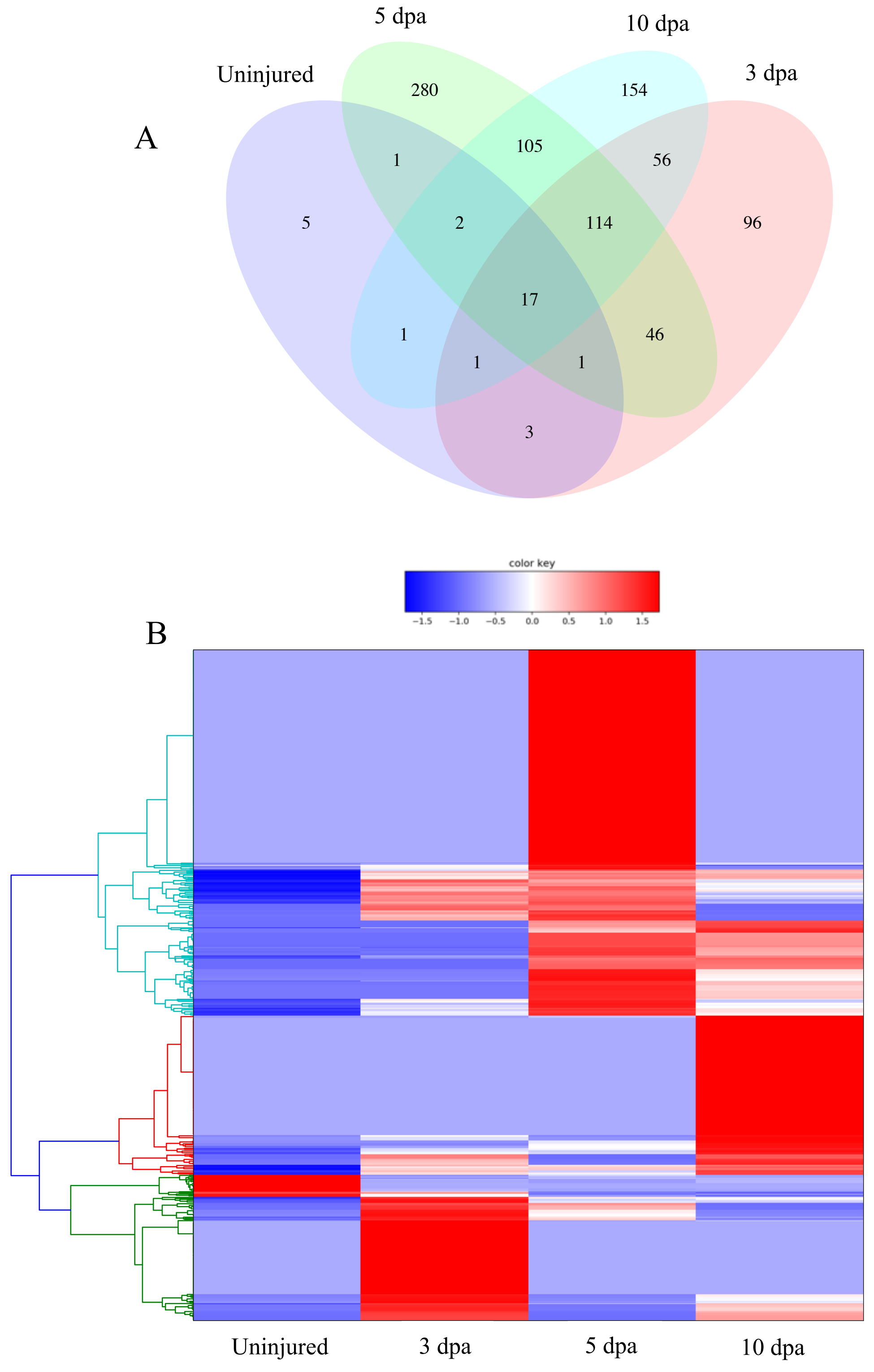

Figure S3 – Bioinformatic analysis of the S-nitrosylome in tailfin regeneration in zebrafish. A-B. Venn diagram and Hierarchical clustering heat map showing changes in the number of S-nitrosylated nuclear peptides, analysed by LC/MS/MS, during the regeneration process compared to uninjured. **C-D.** Venn diagram and Hierarchical clustering heat map showing changes in the number of S-nitrosylated nuclear proteins with a human orthologue during the regeneration process compared to uninjured. Key: dpa – days post-amputation.

C

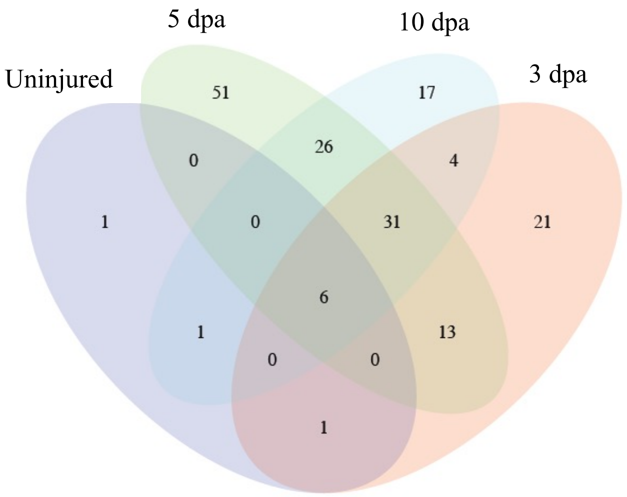
5 dpa
10 dpa
Uninjured
3 dpa
51
17
26
0
4
1
0
31
21
6
1
13
0
0
1

D

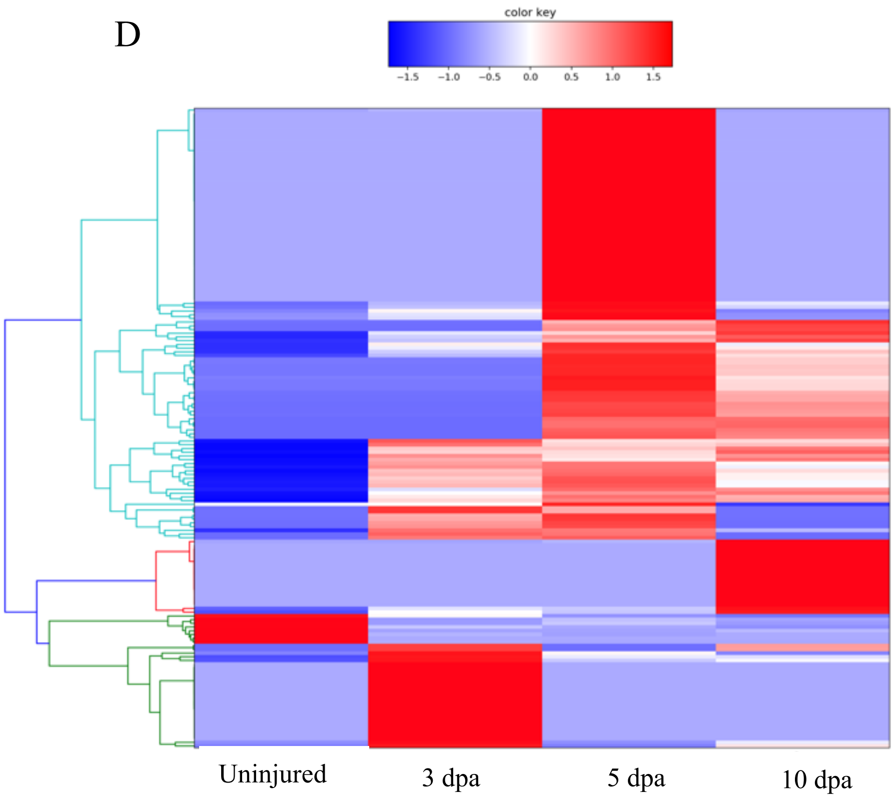
color key
−1.5
−1.0
−0.5
0.0
0.5
1.0
1.5
Uninjured
3 dpa
5 dpa
10 dpa

A

Score	Expect	Method	Identities	Positives	Gaps
1397 bits(3615)	0.0	Compositional matrix adjust.	716/847(85%)	751/847(88%)	38/847(4%)

```
Query  10    GSSSSSGGNGGDRAPETLAAPQAGPAGPS--GPISADVK---KKE--RASPSGEPGGPPL  62
             G+ +  G  GG      +AA  AG +GP+  GP +   +   KKE  RASP   PGG  L
Sbjct  20    GTEAGPGTAGGSENGSEVAAQPAGLSGPAEVGPGAVGERTPRKKEPPRASP---PGG--L  74

Query  63    PHPPGPGGIDQ-----------------DSAEGRRTSRRKRAKVEYREMDESLANLSEDE  105
               PPG  G                    ++ EGRRTSRRKRAKVEYREMDESLANLSEDE
Sbjct  75    AEPPGSAGPQAGPTVVPGSATPMETGIAETPEGRRTSRRKRAKVEYREMDESLANLSEDE  134

Query  106   YYSEEERNAKAEKERKQVIPPPAPPIEEENDSEPEEPSGINGVEGAAFQSRLPHDRMTSQ  165
             YYSEEERNAKAEKE+K       PP     +    EP   +GVEGAAFQSRLPHDRMTSQ
Sbjct  135   YYSEEERNAKAEKEKKLPP---PPPQAPPEEENESEPEEPSGVEGAAFQSRLPHDRMTSQ  191

Query  166   EAACFPDIINGPQHTQKVFLYIRNRTLQLWLDNPKVQLTFEATVQQLEAPYNSDAVLVHR  225
             EAACFPDII+GPQ TQKVFL+IRNRTLQLWLDNPK+QLTFEAT+QQLEAPYNSD VLVHR
Sbjct  192   EAACFPDIISGPQQTQKVFLFIRNRTLQLWLDNPKIQLTFEATLQQLEAPYNSDTVLVHR  251

Query  226   IHSYLERHGFINFGIYKRVKPLPTKKTGKVIVIGAGVSGLAAARQLQSFGMDVTVLESRD  285
             +HSYLERHG INFGIYKR+KPLPTKKTGKVI+IG+GVSGLAAARQLQSFGMDVT+LE+RD
Sbjct  252   VHSYLERHGLINFGIYKRIKPLPTKKTGKVIIIGSGVSGLAAARQLQSFGMDVTLLEARD  311

Query  286   RVGGRVATFRKGNYVADLGAMVVTGLGGNPMAVVSKQVNMELAKIKQKCPLYEANGQAGE  345
             RVGGRVATFRKGNYVADLGAMVVTGLGGNPMAVVSKQVNMELAKIKQKCPLYEANGQA
Sbjct  312   RVGGRVATFRKGNYVADLGAMVVTGLGGNPMAVVSKQVNMELAKIKQKCPLYEANGQA--  369

Query  346   RCTSVPKEKDEMVEQEFNRLLEATSYLSHQLDFNFLNNKPVSLGQALEVVIQLQEKHVKD  405
                 VPKEKDEMVEQEFNRLLEATSYLSHQLDFN LNNKPVSLGQALEVVIQLQEKHVKD
Sbjct  370   ----VPKEKDEMVEQEFNRLLEATSYLSHQLDFNVLNNKPVSLGQALEVVIQLQEKHVKD  425

Query  406   EQIEHWKKIVKTQEELKDLLNKMVTTKEKVKELHQQYKEASEVKPPRDITAEFLVKSKHR  465
             EQIEHWKKIVKTQEELK+LLNKMV  KEK+KELHQQYKEASEVKPPRDITAEFLVKSKHR
Sbjct  426   EQIEHWKKIVKTQEELKELLNKMVNLKEKIKELHQQYKEASEVKPPRDITAEFLVKSKHR  485

Query  466   DLTALCKEYDELVEMQVKLEERLQELEANPPSDVYLSSRDRQILDWHFANLEFANATPLS  525
             DLTALCKEYDEL E Q KLEE+LQELEANPPSDVYLSSRDRQILDWHFANLEFANATPLS
Sbjct  486   DLTALCKEYDELAETQGKLEEKLQELEANPPSDVYLSSRDRQILDWHFANLEFANATPLS  545

Query  526   TLSLKHWDQDDDFEFTGSHLTVRNGYSCVPVALAEGLDIKLNTAVRQVRYTSSGCEVIAV  585
             TLSLKHWDQDDDFEFTGSHLTVRNGYSCVPVALAEGLDIKLNTAVRQVRYT+SGCEVIAV
Sbjct  546   TLSLKHWDQDDDFEFTGSHLTVRNGYSCVPVALAEGLDIKLNTAVRQVRYTASGCEVIAV  605

Query  586   NTRSTTQTFIYKCDAVLCTLPLGVMKQQPPAVQFVPPLPEWKTAAIQRMGFGNLNKVVLC  645
             NTRST+QTFIYKCDAVLCTLPLGV+KQQPPAVQFVPPLPEWKT+A+QRMGFGNLNKVVLC
Sbjct  606   NTRSTSQTFIYKCDAVLCTLPLGVLKQQPPAVQFVPPLPEWKTSAVQRMGFGNLNKVVLC  665

Query  646   FDRVFWDPSVNLFGHVGSTTASRGELFLFWNLYKAPILLALMAGEAAGIMENISDDVIVG  705
             FDRVFWDPSVNLFGHVGSTTASRGELFLFWNLYKAPILLAL+AGEAAGIMENISDDVIVG
Sbjct  666   FDRVFWDPSVNLFGHVGSTTASRGELFLFWNLYKAPILLALVAGEAAGIMENISDDVIVG  725

Query  706   RCLAILKGIFGSSAVPQPKETVVSRWRADPWARGSYSYVAAGSSGNDYDLMAQPITPGPA  765
             RCLAILKGIFGSSAVPQPKETVVSRWRADPWARGSYSYVAAGSSGNDYDLMAQPITPGP+
Sbjct  726   RCLAILKGIFGSSAVPQPKETVVSRWRADPWARGSYSYVAAGSSGNDYDLMAQPITPGPS  785

Query  766   IPGASQPVPRLFFAGEHTIRNYPATVHGALLSGLREAGRIADQFLGAMYTMPRQATANPN  825
             IPGA QP+PRLFFAGEHTIRNYPATVHGALLSGLREAGRIADQFLGAMYT+PRQAT
Sbjct  786   IPGAPQPIPRLFFAGEHTIRNYPATVHGALLSGLREAGRIADQFLGAMYTLPRQATPGVP  845

Query  826   PQPSPSI  832
              Q SPS+
Sbjct  846   AQQSPSM  852
```

B

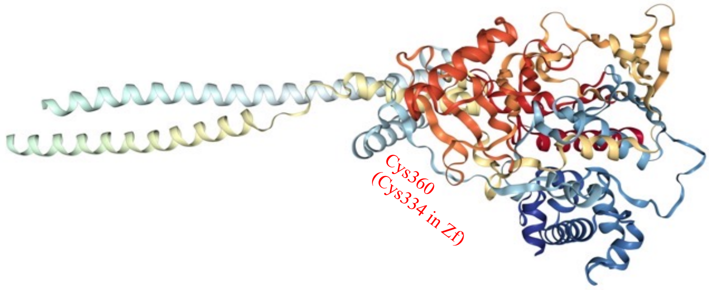

Figure S4 – Similarities between zebrafish and human Kdm1a. A. Comparison of Kdm1a (also known as Lsd1) protein in Zebrafish (Danio rerio, Uniprot F6NIA2) and Human (Homo sapiens, Uniprot O60341), analysed using BLAST (https://blast.ncbi.nlm.nih.gov). The red rectangle encloses the cysteine (position 334 in zebrafish and 360 in human). **B.** Crystal structure of Human Kdm1a (Protein Data Bank no. 6NQM), showing the position of cysteine 360.

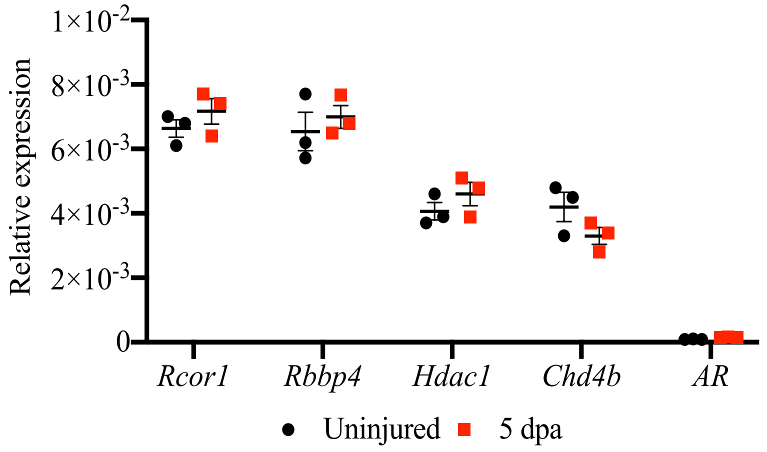

Figure S5 – Expression of *Rcor1, Rbbp4, Hdac1, Chd4b* and *Androgen receptor* (*ar*) genes. Bar graph showing real time PCR for the expression of these genes in zebrafish tailfin at 5 days post-amputation (dpa). N=3 biological replicates. Two-tailed t-test was used to compare the means. No statistical significance was observed. N=3 biological replicates. Data are presented as mean values +/- SEM.

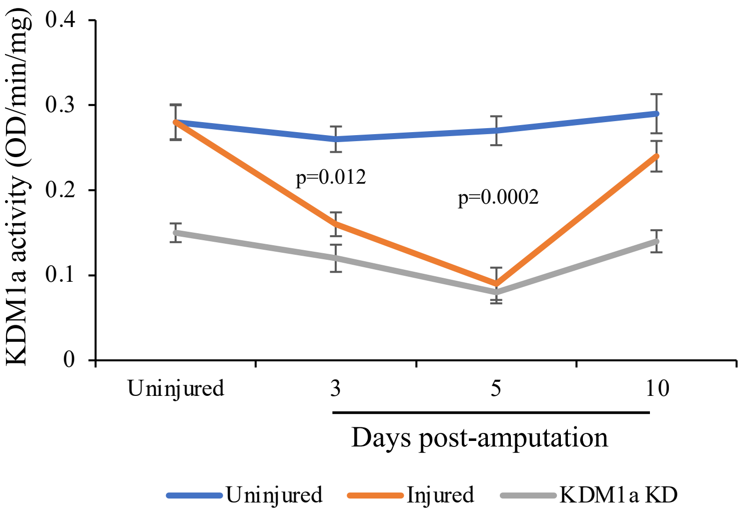

Figure S6 – Kdm1a activity. Analysis of Kdm1a demethylase enzymatic activity, expressed as optical density (OD)/min/mg, during the regeneration and following *kdm1a* KD. N=3 biological replicates. Two-way ANOVA test followed by Bonferroni's multiple comparisons test. N=3 biological replicates. Data are presented as mean values +/- SEM.

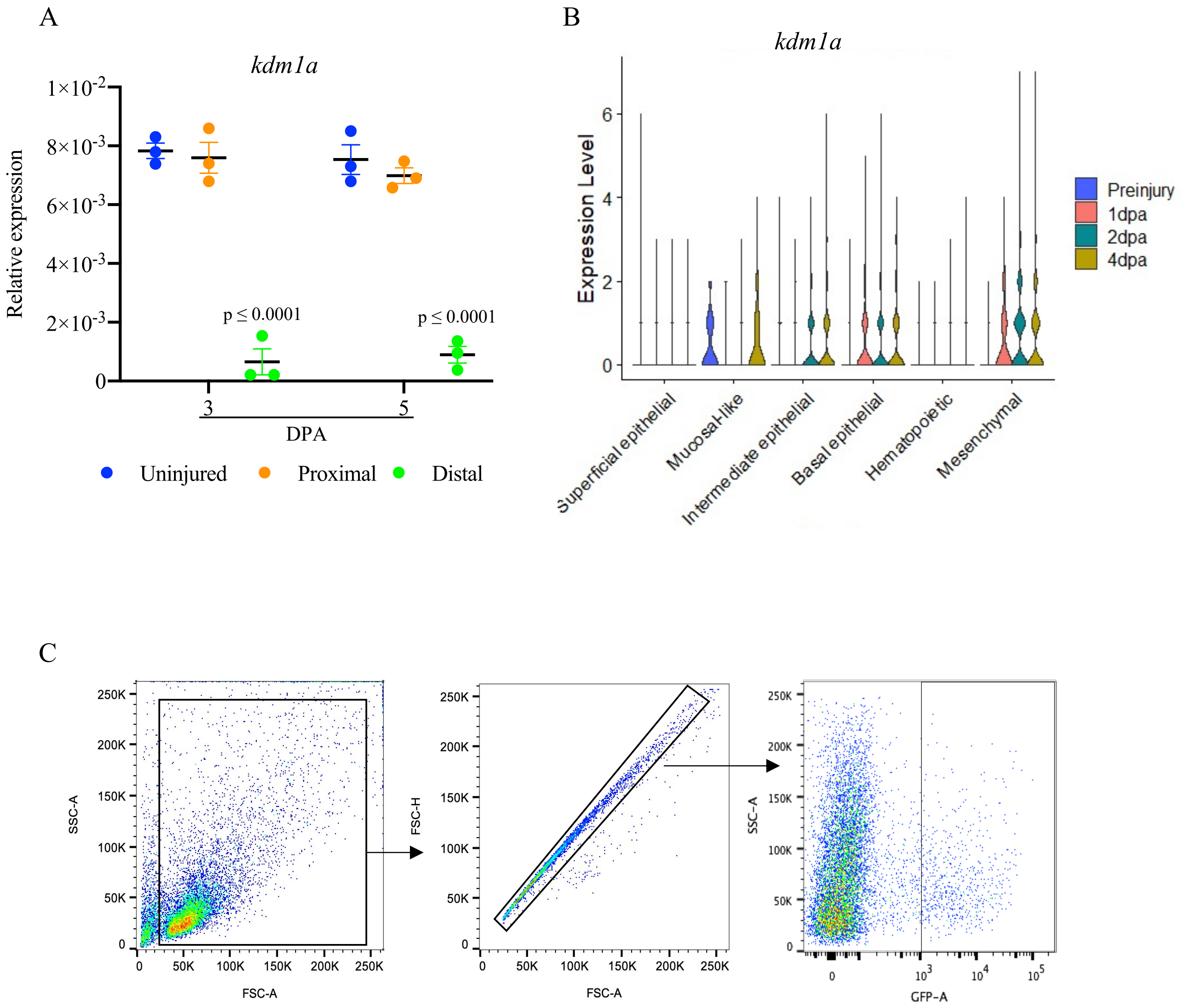

Figure S7 – A. Bar graph showing real-time PCR for the *kdm1a* in the whole, proximal and distal area of the zebrafish tailfin at 3 and 5 days post-amputation (dpa). Two-way ANOVA test followed by Bonferroni's multiple comparisons test. N=3 biological replicates. Data are presented as mean values +/- SEM. **B.** Violin plot of *kdm1a* expression across cell types during regeneration of zebrafish tailfin, obtained from single-cell datasets by Hou et al. (22). ****P*<0.001. ANOVA test followed by Bonferroni post hoc test was used to compare the means. **C.** Gating strategy for detection of GFP+ (endothelial) cells from Tg(fli1:EGFP)y1 zebrafish embryos. The same gating strategy was applied on all FACS analyses.

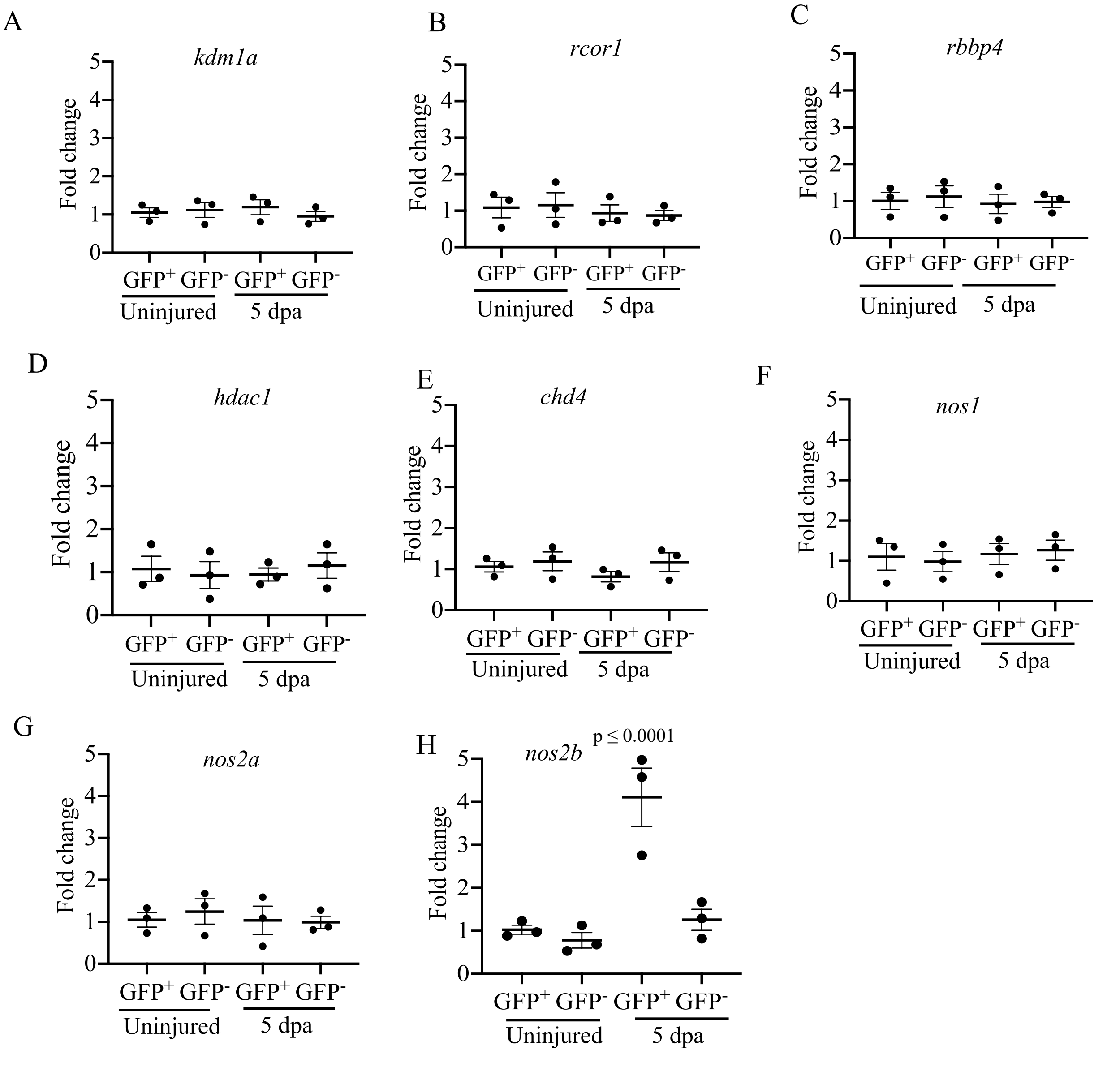

Figure S8 – Expression of gene implicated in Kdm1a function. Real time PCR was used to perform the analysis in GFP$^+$ (endothelial) and GFP$^-$ (non-endothelial) cells isolated from zebrafish *Tg(fli1:EGFP)*^{y1} uninjured (control) and injured tailfin at 5 days post-amputation (dpa). Among the *nos* genes, the expression of *nos2b* was significantly increased in GFP$^+$ of injured tailfin compared to control. The expression of other Nos and Kdm1a and its interacting proteins Rcor1 Rbbp4, Hdac1 and Chd4 was unchanged among the groups. Two-way ANOVA test followed by Bonferroni's multiple comparisons test. N=3 biological replicates. Data are presented as mean values +/- SEM.

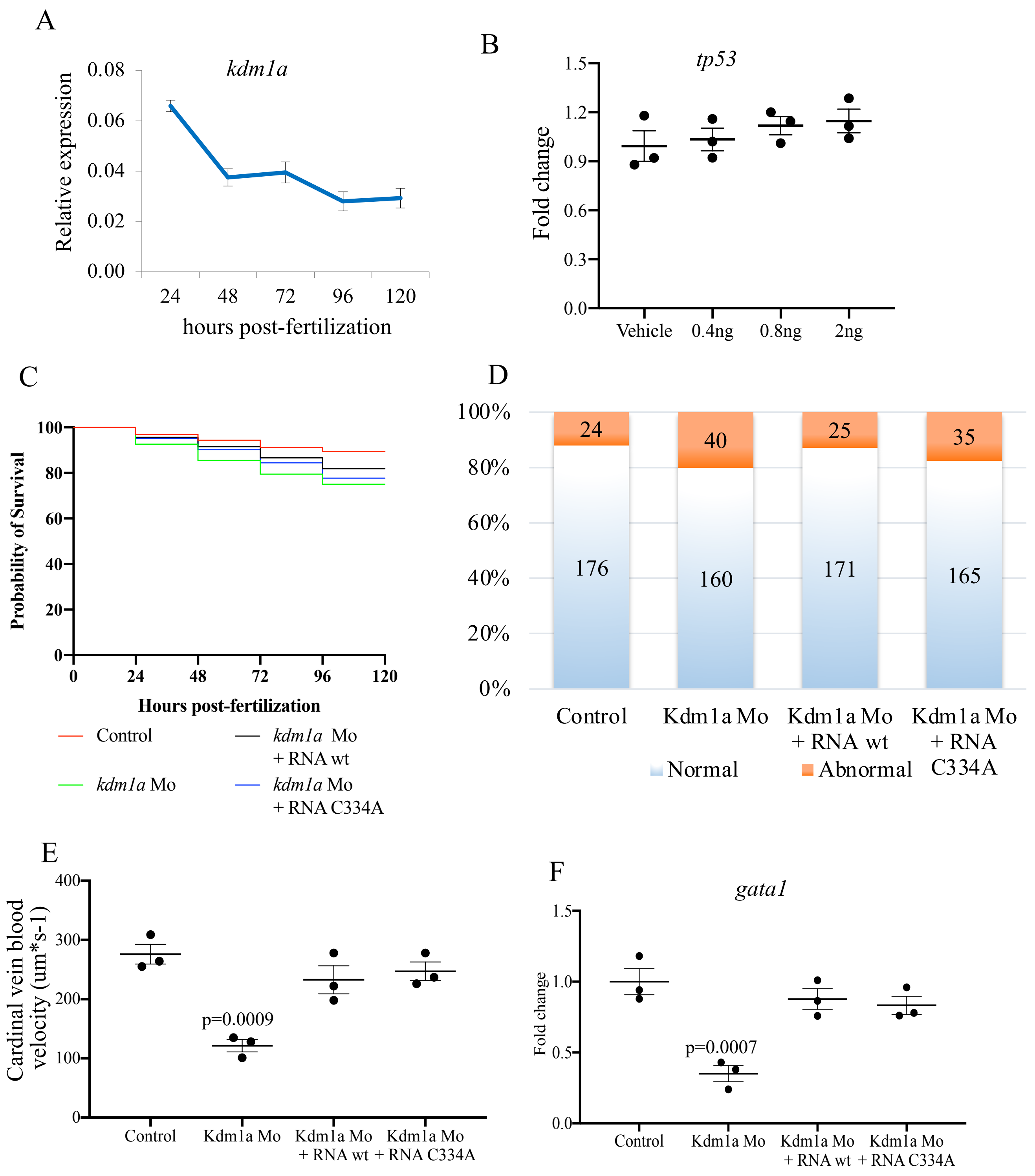

Figure S9 – Effects of *kdm1a* knockdown in zebrafish embryos and rescue by wild-type and C334A (mutant) mRNA. A. Real time PCR analysis for *kdm1a* gene expression during zebrafish development. **B.** Real time PCR for of *tp53* gene expression in zebrafish embryos at increasing dose of *kdm1a* morpholino. **C.** Kaplan-Meyer survival curve showing the survival rate following injection of *kdm1a* Mo and mutant or wild type mRNA. **D.** Bar graph showing the analysis of phenotypic and functional features. Normal - embryos not showing any abnormal features, Abnormal - embryos showing one or more of the features: reduced length, curved body, chorionated, edema mild, edema severe, reduced swim. **E.** Analysis of blood velocity in the caudal vein following injection of *kdm1a* Mo and mutant or wild type mRNA. **F.** Real time PCR for of *gata1*. PCR data were ormalized to β-actin. One-way ANOVA followed by Bonferroni's multiple comparisons test was used in **B**, **E** and **F**, p values vs control. N=3 biological replicates. Data are presented as mean values +/- SEM.

Table S1 – List of S-nitrosylated proteins selected for deep mass-spec analysis.
Kdm1a, marked in red text, has been selected for further studies.

Gene	Gene full name	Source
aldoa	aldolase a, fructose-bisphosphate, a	ZFIN;Acc:ZDB-GENE-030131-8369
atrx	alpha thalassemia/mental retardation syndrome X-linked homolog	ZFIN;Acc:ZDB-GENE-030912-11
Bub3	BUB3 mitotic checkpoint protein	ZFIN;Acc:ZDB-GENE-041010-210
zcchc13	CCHC-type zinc finger, nucleic acid binding protein b	ZFIN;Acc:ZDB-GENE-030131-7782
cpsf2	cleavage and polyadenylation specific factor 2	ZFIN;Acc:ZDB-GENE-040718-79
ddx5	DEAD (Asp-Glu-Ala-Asp) box helicase 5	ZFIN;Acc:ZDB-GENE-030131-925
dhx9	DEAH (Asp-Glu-Ala-His) box helicase 9	ZFIN;Acc:ZDB-GENE-070912-171
eef2	eukaryotic translation elongation factor 2, like 2	ZFIN;Acc:ZDB-GENE-030131-8112
huwe1	HECT, UBA and WWE domain containing 1	ZFIN;Acc:ZDB-GENE-081104-387
hnrnpab	heterogeneous nuclear ribonucleoprotein A/Bb	ZFIN;Acc:ZDB-GENE-040426-2516
hnrnpk	heterogeneous nuclear ribonucleoprotein K	ZFIN;Acc:ZDB-GENE-040426-1926
Ac008982.1	heterogeneous nuclear ribonucleoprotein L2	ZFIN;Acc:ZDB-GENE-040426-2707
hnrnpm	heterogeneous nuclear ribonucleoprotein M	ZFIN;Acc:ZDB-GENE-030131-6898
hexim1	hexamethylene bis-acetamide inducible 1	ZFIN;Acc:ZDB-GENE-030131-4637
kdm1a	lysine (K)-specific demethylase 1a	ZFIN;Acc:ZDB-GENE-030131-7828
mphosph8	M-phase phosphoprotein 8	ZFIN;Acc:ZDB-GENE-050309-191
nsun2	NOP2/Sun RNA methyltransferase family, member 2	ZFIN;Acc:ZDB-GENE-030131-4017
nop56	NOP56 ribonucleoprotein homolog	ZFIN;Acc:ZDB-GENE-040109-1
nop58	NOP58 ribonucleoprotein homolog	ZFIN;Acc:ZDB-GENE-040426-2140
ncl	nucleolin	ZFIN;Acc:ZDB-GENE-030131-6986
parp1	poly (ADP-ribose) polymerase 1	ZFIN;Acc:ZDB-GENE-030131-3955
pa2g4	proliferation-associated 2G4, b	ZFIN;Acc:ZDB-GENE-030131-2182
rif1	replication timing regulatory factor 1	ZFIN;Acc:ZDB-GENE-030131-3406
rbm4	RNA binding motif protein 4.1	ZFIN;Acc:ZDB-GENE-030131-3902
snrnp200	small nuclear ribonucleoprotein 200	ZFIN;Acc:ZDB-GENE-081105-64
sod1	superoxide dismutase 1, soluble	ZFIN;Acc:ZDB-GENE-990415-258
thoc1	THO complex 1	ZFIN;Acc:ZDB-GENE-030826-9
tcea1	transcription elongation factor A (SII), 1	ZFIN;Acc:ZDB-GENE-030131-8049
tpt1	tumor protein, translationally-controlled 1	ZFIN;Acc:ZDB-GENE-990603-10
znf346	zinc finger protein 346	ZFIN;Acc:ZDB-GENE-070209-152
znf865	zinc finger protein 865	ZFIN;Acc:ZDB-GENE-060503-896

Table S2 – List of primers.

Gene	Primers
kdm1a (NM_001242995.1)	Forward GAAGTAAAGCCACCCAGAGAC<br>Reverse CATCTCCACCAGCTCATCATAC
nos1 (NM_131660.1)	Forward GAACGGAAGTCCCTCCAAAT<br>Reverse TTACTGGAGCTGTGGTGTAAAG
nos2a (NM_001104937.1)	Forward CACTGTCTGCTCTTCACTCTAAA<br>Reverse TTCAGCTCCACCAGGATAGTA
nos2b (NM_001113501.1)	Forward CCAGGCTCTCACCTACTATCT<br>Reverse TCCTCCTGTCTGGCTATCTT
hdac1 (NM_173236.1)	Forward GTCCTGTCTTTGACGGCTTAT<br>Reverse GCAATGTCTGTCTGCTGTTTG
rcor1 (NM_001080041.1)	Forward CACTGCCAACAGTAGAGTTTCT<br>Reverse TGGCTTTAGGGTGGGTTTATG
rbbp4 (NM_212595.1)	Forward CTGGGACACTCGTTCCAATAA<br>Reverse ACTCGCTGTAAGGGTTGAAG
chd4b (XM_680607.9), ortholog to human *Chd4*	Forward CTCCTGGCTGGGATCATATTC<br>Reverse CCCTCTGCTCATTTCTCCTTTA
gata1 (NM_131234.2)	Forward GATGAGCATGTAGGAGCGTATT<br>Reverse CAAAGTGGTAGAGGAGTGTAAGG
ar - Androgen receptor (NM_001083123.1)	Forward AGCGACGAGACTGAACAATAC<br>Reverse GGGAGAGACGAGACTGAGATAG
relA (P65) (NM_001001839.2)	Forward CTGAAGATCTGCCGTGTCAATA<br>Reverse GCACCTCAATGTCCTCTTTCT
kdr (NM_001024653.2)	Forward CTGAAACCCGGCTCACTATATC<br>Reverse GCTATCCCAGATCACCTCTTTC
mmp2 (NM_198067.1)	Forward CCAGTACCCTGGAAAGAGAATATC<br>Reverse TTGGTCTTGTGGAAGGAGTAAG
angpt2 (NM_131814.1)	Forward CGTCAGGAGAACACTGGTTAG<br>Reverse CTGGTGTTCCTCTGCATCTT
fgf2 (NM_212823.2)	Forward ACAGACGGCGGGTTTATTT<br>Reverse AGATCATATCGGTGGGATTTGG
tek (NM_131461.1)	Forward CTGTGGGAAGTGGTCAGTTTAG<br>Reverse TCGTCACAGTTCAGTGGTTTC
cdh5 (NM_001003983.1)	Forward CTCAGCTTCTCTCCTCAACATAC<br>Reverse TGAAGAGGTCTGTGCCATTTAG
cd31 (NM_001113799.1)	Forward GCGTGCAGAAGAAGGTAGAA<br>Reverse ATCTGATGGAGGTGATGGTAAAG
vegfaa (NM_131408.3)	Forward GAGCTGCTGGTAGACATCATC<br>Reverse TTCGAGCGCCTCATCATTAC
tie1 (NM_001346150.1)	Forward CCTGAAGCCCTCAACCAAATA<br>Reverse CTTGCTGAGTGGTGGGAATAA
tbx20 (NM_131506.2)	Forward CCTGACACTGCAGAGTGATAC<br>Reverse GGTCGTCCGGTACTGTTATTT
actb1 (NM_131031.2)	Forward GATCTGGCATCACACCTTCTAC<br>Reverse CACCAGAGTCCATCACAATACC
