# Supplementary material for: Nuclear S-nitrosylation impacts tissue regeneration in zebrafish
Source: Nat Commun. 2021 Nov 1;12:6282. doi: 10.1038/s41467-021-26621-0 (PMC8560954; doi:10.1038/s41467-021-26621-0)
Supplement: Supplementary file 3 — Description of Additional Supplementary Files [file 41467_2021_26621_MOESM3_ESM.pdf]

Description of Additional Supplementary Data

File name: Supplementary Data 1

Description: List of nuclear S-nitrosylated proteins at different time-points during zebrafish tailfin regeneration.
