# Supplementary material for: Nuclear S-nitrosylation impacts tissue regeneration in zebrafish
Source: Nat Commun. 2021 Nov 1;12:6282. doi: 10.1038/s41467-021-26621-0 (PMC8560954; doi:10.1038/s41467-021-26621-0)
Supplement: Supplementary file 5 — Reporting Summary [file 41467_2021_26621_MOESM5_ESM.pdf]

nature research

Corresponding author(s): Gianfranco Matrone

Last updated by author(s): Sep 18, 2021

Reporting Summary

Nature Research wishes to improve the reproducibility of the work that we publish. This form provides structure for consistency and transparency in reporting. For further information on Nature Research policies, see our Editorial Policies and the Editorial Policy Checklist.

Statistics

For all statistical analyses, confirm that the following items are present in the figure legend, table legend, main text, or Methods section.

n/a	Confirmed	
☐	☒	The exact sample size (*n*) for each experimental group/condition, given as a discrete number and unit of measurement
☐	☒	A statement on whether measurements were taken from distinct samples or whether the same sample was measured repeatedly
☐	☒	The statistical test(s) used AND whether they are one- or two-sided<br>*Only common tests should be described solely by name; describe more complex techniques in the Methods section.*
☒	☐	A description of all covariates tested
☒	☐	A description of any assumptions or corrections, such as tests of normality and adjustment for multiple comparisons
☐	☒	A full description of the statistical parameters including central tendency (e.g. means) or other basic estimates (e.g. regression coefficient) AND variation (e.g. standard deviation) or associated estimates of uncertainty (e.g. confidence intervals)
☐	☒	For null hypothesis testing, the test statistic (e.g. *F*, *t*, *r*) with confidence intervals, effect sizes, degrees of freedom and *P* value noted<br>*Give P values as exact values whenever suitable.*
☒	☐	For Bayesian analysis, information on the choice of priors and Markov chain Monte Carlo settings
☒	☐	For hierarchical and complex designs, identification of the appropriate level for tests and full reporting of outcomes
☐	☒	Estimates of effect sizes (e.g. Cohen's *d*, Pearson's *r*), indicating how they were calculated

Our web collection on statistics for biologists contains articles on many of the points above.

Software and code

Policy information about availability of computer code

Data collection	Proteome Discoverer, version 2.4;<br>Zebrafish UniProt database (Danio rerio; UP000000437);<br>Ingenuity pathway analysis.
Data analysis	Leica LAS X;<br>Image J;<br>Prism 8;<br>FlowJo 10.

For manuscripts utilizing custom algorithms or software that are central to the research but not yet described in published literature, software must be made available to editors and reviewers. We strongly encourage code deposition in a community repository (e.g. GitHub). See the Nature Research guidelines for submitting code & software for further information.

Data

Policy information about availability of data

All manuscripts must include a data availability statement. This statement should provide the following information, where applicable:

- Accession codes, unique identifiers, or web links for publicly available datasets
- A list of figures that have associated raw data
- A description of any restrictions on data availability

TMT-labelled S-nitrosylated protein analysis of mass spectrometry data have been deposited to the ProteomeXchange Consortium (http://proteomecentral.proteomexchange.org/cgi/GetDataset?ID=PXD017883) via the MASSIVE repository (MSV000085055) with the dataset identifier PXD017883. Furthermore, a full list of the S-nitrosylated proteins derived from the mass-spec is included in this manuscript as Dataset S1.

Zebrafish UniProt database (Danio rerio; UP000000437).
Gene Expression Omnibus (GEO) database, accession GSE137971, was used for single cell sequencing analysis of publicly available dataset. Protein Data bank (https://www.ebi.ac.uk/pdbe/entry/pdb/6nqm) was used for protein crystal structure analysis. Protein Blast (https://blast.ncbi.nlm.nih.gov/Blast.cgi) was used to compare protein sequences.

Field-specific reporting

Please select the one below that is the best fit for your research. If you are not sure, read the appropriate sections before making your selection.

☒ Life sciences ☐ Behavioural & social sciences ☐ Ecological, evolutionary & environmental sciences

For a reference copy of the document with all sections, see nature.com/documents/nr-reporting-summary-flat.pdf

Life sciences study design

All studies must disclose on these points even when the disclosure is negative.

Sample size	Samples size were not predetermined using statistical methods but were selected on the basis of our previous studies that included similar experiments using the zebrafish (Matrone et al., PNAS, 2021; Matrone et al., ATVB, 2017). In particular, for mass-spec experiment the sample size was chosen based on pilot experiments to assess the amount of nuclear proteins obtained by each adult zebrafish tailfin tissue and considering that the mass-spec run required about 250 ug of S-nitrosopeptides. Accordingly, at least n=15 adult fish per group were required for mass-spec analysis. Also, based on our previous experience with Fli1+ cell isolated form adult zebrafish, n=10 adult fish were used to isolate endothelial cells from the tailfin for each group for FACS and sorting analysis and for subsequent proteins and RNA purification. For experiments involving zebrafish embryos, at least n= 6 embryos were included in each group.
Data exclusions	No data were excluded for the analyses.
Replication	All experiments were reliably reproduced. N=3 independent biological replicates were performed for each assay as also specified in the respective legends. An assessment of intra-observer variation of the measurements for each replicate was undertaken and assessed by repeating the measure on two separate occasions.
Randomization	Zebrafish enrolled in each experiment were first checked for any health issue. All healthy fish were placed in a 10 L tank and randomly collected by the investigator with a net and placed in a smaller tank.
Blinding	Mass-spec analysis and FACS analysis and sorting experiments were performed by investigators working at the instruments that were not aware of the experimental groups provided by the investigator that prepared these samples. For all other experiments, as an internal control to confirm our interpretations, other members of the lab not involved in the experiments and not aware of the experimental conditions were asked to offer their interpretation of the data.

Reporting for specific materials, systems and methods

We require information from authors about some types of materials, experimental systems and methods used in many studies. Here, indicate whether each material, system or method listed is relevant to your study. If you are not sure if a list item applies to your research, read the appropriate section before selecting a response.

Materials & experimental systems

n/a	Involved in the study	
☐	☒	Antibodies
☒	☐	Eukaryotic cell lines
☒	☐	Palaeontology and archaeology
☐	☒	Animals and other organisms
☒	☐	Human research participants
☒	☐	Clinical data
☒	☐	Dual use research of concern

Methods

n/a	Involved in the study	
☒	☐	ChIP-seq
☐	☒	Flow cytometry
☒	☐	MRI-based neuroimaging

Antibodies

Antibodies used

anti-KDM1a (1:200, Thermo Fisher Scientific, San Jose, CA, cat. PA1-41697), western blot and immunoprecipitation.
anti-iNOS (1:200, BD Transduction Laboratories, San Jose, CA, cat. 610432), western blot.
anti-beta-tubulin (1:500, Abcam, Cambridge, UK, cat. ab6046), western blot.
anti-Histone H3 (1:500, Abcam, Cambridge, UK, cat. ab1791), western blot.
anti-Histone H3 (unmodified Lys4) (1:500, Merck Millipore, Massachusetts, USA, cat. 05-1341), western blot.
anti-monomethyl-Histone H3 (Lys4) (1:500, Merck Millipore, Massachusetts, USA, cat. 07-436), western blot.
anti-dimethyl-Histone H3 (Lys4) (1:500, Merck Millipore, Massachusetts, USA, cat. 04-790), western blot.
anti-monomethyl-Histone H3 (Lys9) (1:500, Merck Millipore, Massachusetts, USA, cat. ABE101), western blot.
anti-dimethyl-Histone H3 (Lys9) (1:500, Merck Millipore, Massachusetts, USA, cat. 07-212), western blot.

	anti-RCOR1 (1:200, Invitrogen, Carlsbad, CA, cat. PA5-41564), western blot.<br>anti-HDAC1 (1:200, Abcam, Cambridge, UK, cat. ab33278), western blot.<br>anti-RBBP4 (1:200, Biorbyt, Cat. orb583248), western blot.<br>anti-CHD4 (1:200, Biorbyt, Cambridge, UK, cat. orb575051), western blot.<br>anti-TMT (1:200, Thermo Fisher Scientific, San Jose, CA, cat. 90075), western blot.<br>HRP-anti-mouse (1:2000, Santa Cruz Biotechnology, Dallas, USA, SC-2005), western blot.<br>HRP-anti-rabbit (1:2000, Santa Cruz Biotechnology, Dallas, USA, SC-2004), western blot.
Validation	Validation was performed by using different dose of primary antibodies (1:100 -1:1000) to select the optimal concentration to use for the experiments.<br>anti-KDM1a (Thermo Fisher Scientific, PA1-41697), manufacturer's validation.<br>anti-iNOS (1:200, BD Transduction Laboratories, San Jose, CA, cat. 610432), cited in Diaz-Casado M et al., J Pineal Research, 2016 (https://doi.org/10.1111/jpi.12332) and further validated in our lab.<br>anti-beta-tubulin (Abcam, ab6046), predicted to react with zebrafish by the manufacturer and validated in our lab.<br>anti-Histone H3 (Abcam, ab1791), predicted to react with zebrafish by the manufacturer and validated in our lab.<br>anti-Histone H3 (unmodified Lys4) (Merck Millipore, 05-1341), broad species cross-reactivity expected according to the manufacturer and validated in our lab.<br>anti-monomethyl-Histone H3 (Lys4) (Merck Millipore, 07-436), broad species cross-reactivity expected according to the manufacturer and validated in our lab.<br>anti-dimethyl-Histone H3 (Lys4) (Merck Millipore, 04-790), broad species cross-reactivity expected according to the manufacturer and validated in our lab.<br>anti-monomethyl-Histone H3 (Lys9) (Merck Millipore, ABE101), broad species cross-reactivity expected according to the manufacturer and validated in our lab.<br>anti-dimethyl-Histone H3 (Lys9) (Merck Millipore, 07-212), broad species cross-reactivity expected according to the manufacturer and validated in our lab.<br>anti-RCOR1 (Invitrogen, PA5-41564), predicted to react with zebrafish by the manufacturer and validated in our lab.<br>anti-HDAC1 (Abcam, ab33278), manufacturer's validation.<br>anti-RBBP4 (Biorbyt, orb583248), manufacturer's validation.<br>anti-CHD4 (Biorbyt, orb575051), manufacturer's validation.<br>anti-TMT (Thermo Fisher Scientific, cat. 90075).<br>HRP-anti-mouse (1:2000, Santa Cruz Biotechnology, Dallas, USA, SC-2005), western blot.<br>HRP-anti-rabbit (1:2000, Santa Cruz Biotechnology, Dallas, USA, SC-2004), western blot. manufacturer's validation

Animals and other organisms

Policy information about studies involving animals; ARRIVE guidelines recommended for reporting animal research

Laboratory animals	This work involved the use of zebrafish (Danio rerio) at larval stage from 1 to 5 days post-fertilization, and adult 6-9 months old with equal number of female and males per group. Tg(nfkb:GFP), Tg(Fli1:EGFP) and wik (wild-type) were the lines used.
Wild animals	No wild animals were used in the study.
Field-collected samples	No field collected samples were used in the study.
Ethics oversight	All experiments were carried out in accordance with the recommendations of the Institutional Animal Care and Use Committee at the Houston Methodist Research Institute, and with the United Kingdom Animals (Scientific Procedures) Act 1986 at the Queens Medical Research Institute research facilities.

Note that full information on the approval of the study protocol must also be provided in the manuscript.

Flow Cytometry

Plots

Confirm that:

☒ The axis labels state the marker and fluorochrome used (e.g. CD4-FITC).

☒ The axis scales are clearly visible. Include numbers along axes only for bottom left plot of group (a 'group' is an analysis of identical markers).

☒ All plots are contour plots with outliers or pseudocolor plots.

☒ A numerical value for number of cells or percentage (with statistics) is provided.

Methodology

Sample preparation	Cell suspensions were derived by enzymatic disaggregation of the adult Tg(fli1:EGFP) zebrafish tailfin, then placed in PBS and analysed using a BD FACS Aria (BD Biosciences). DAPI staining was used to identify viable single cells. The number of viable cells was confirmed under fluorescence stereomicroscope (Leica M205) by using a Neubauer chamber.
Instrument	BD FACS Aria (BD Biosciences)
Software	BD FACS Aria software and FlowJo 10 (Becton and Dickinson).

Cell population abundance	At least 10,000 of GFP- or GFP+ cells (Ex: 488 nm; Em: 530 nm)
Gating strategy	FSC-H and FSC-A were used to select cell singlets. Wild-type (Wik) zebrafish was used to set the gate between GFP- (i.e. Fli1-) and GFP+ (Fli1+) cell populations.

☒ Tick this box to confirm that a figure exemplifying the gating strategy is provided in the Supplementary Information.
